# Evaluating Statin Knowledge-Perceptions and Receptivity Following a Comprehensive Lifestyle Modification Program

**DOI:** 10.1177/15598276231163129

**Published:** 2023-03-16

**Authors:** Alaina Pupulin, Jillian Ball, Ravi Bajaj, David A. Alter

**Affiliations:** Faculty of Medicine, 12365 University of Ottawa, Ottawa, ON, Canada (AP); Faculty of Art and Science, 98586 University of Toronto, Toronto, ON, Canada (JB); Faculty of Medicine, 12366 University of Toronto, Toronto, ON, Canada (RB, DAA); Institute of Health Policy, Management, and Evaluation, University of Toronto, Toronto, ON, Canada (DAA); Rehabilitation Institute-University Health Network, University of Toronto, Toronto, ON, Canada (DAA); Institute for Clinical Evaluative Sciences, Toronto, ON, Canada (DAA)

**Keywords:** statin knowledge-perceptions, statin receptivity, lifestyle modification, medication perspectives

## Abstract

**Background:**

Though clinical guidelines for cholesterol-lowering therapies advocate for both a trial of lifestyle modification and the initiation of statin medication when appropriate, the extent to which lifestyle modification may alter a patient’s knowledge-perceptions and receptivity towards statins remains unclear.

**Methods:**

Following completion of a 6-month comprehensive lifestyle modification program, perceived changes in knowledge and receptivity towards statins were examined across prespecified subgroups of age, sex, and statin eligibility using a mixed-methods questionnaire. Quantitative and qualitative analyses incorporated binomial tests, McNemar’s test, and thematic analyses.

**Results:**

Among 192 patients who completed the program and exit questionnaire between December 15, 2020 and July 2, 2021, 88.4% of patients indicated a perceived improvement in cholesterol and/or statin knowledge (P < .*0001*). 48.2% of patients acknowledged that their receptivity towards taking statins increased (P = .*61*). Changes in receptivity were attributed to several identified program themes including improvements in health knowledge and awareness, motivation and empowerment. Patients who noted improvements in their mental health also reported significantly increased receptivity towards statins (P < .*001*).

**Conclusions:**

Patients’ perceived knowledge and receptivity towards statins may improve following participation in a comprehensive therapeutic lifestyle modification program. Future research must evaluate the impact of these programs on statin uptake, compliance and outcomes.

“In this study, statin receptivity was reported to be significantly higher among those individuals who also reported high overall and mental health status.”

## Brief Summary

Despite clinical evidence demonstrating that statins improve cardiovascular outcomes among at-risk populations, a sizable minority of patients eligible for statins refuse to initiate or sustain therapy. The extent to which knowledge and behavioral changes associated with comprehensive lifestyle modification programs can alter a patient’s knowledge and receptivity to statins remains unclear. Our study demonstrates that patients’ perceived knowledge and receptivity towards statins may improve following their participation in a comprehensive therapeutic lifestyle program.

## Background

Clinical evidence has demonstrated that statin medication reduces the risk of cardiovascular disease in primary and secondary prevention patients and improves cardiovascular outcomes among at-risk populations.^1^ Despite these proven benefits, significant gaps in the utilization and/or adherence to such medications still exist.^2^ For example, a study released by Statistics Canada in 2016 reported that although 2.8 million Canadian adults were treated with statins, 6.5 million Canadian adults (about 1 in 4) were recommended statins under the Canadian Cardiovascular Society (CCS) guidelines but did not undergo treatment.^3^ Patient receptivity to statin treatment remains a challenge in optimizing the real-world utilization of this evidence-based therapy.^4^ The reasons for the lack of statin receptivity among such populations remain multifactorial. Such reasons may include misleading information about the risk-benefit trade-offs of statins, as well as the lack of time physicians spend on educating and counseling patients.^5^ Moreover, studies have shown that patient factors, such as variations in health status and/or mental health may also impact medication hesitancy or receptivity.^6-10^ Because of these reasons, many at-risk individuals prefer to opt for lifestyle modification to manage their cholesterol.^11^ Several current clinical guidelines, including the CCS guidelines for the management of dyslipidemia, continue to advocate for both a trial of lifestyle modification followed by the initiation of a statin when appropriate.^12,13^ Though the impact of medication-taking behaviors on adherence to lifestyle changes has been previously studied, the extent to which a trial of lifestyle modification may actually alter a patient’s receptivity to statins has yet to be investigated.^14^

Accordingly, the primary objective of this study was to evaluate knowledge-perceptions and receptivity surrounding the use of statin medication following a comprehensive lifestyle modification program. Given evidence suggesting self-perceived health status may impact medical decision-making behavior,^6-10^ a secondary objective of this paper was to explore the potential relationship between self-perceived health status and statin receptivity following program completion.

## Methods

### Study Setting - Comprehensive Lifestyle Modification Program

This prospective quality improvement initiative incorporated an ambulatory-based telemedicine lifestyle intervention program (My Heart Fitness^TM^). The program requires a referral from a primary care or specialty care physician, and is fully integrated within Ontario’s publicly funded health-care system. Eligible patients are any adult (18 years of age or older) who is felt to be in need of a preventative lifestyle program because they are deemed at risk for cardiovascular disease (one or more vascular risk-factors) or already have established vascular disease (cardiovascular, peripheral vascular, or cerebrovascular disease). Given the broad applicability of prevention and educational counseling for lifestyle modification, exclusion criteria based on prespecified risk-factors, health-status, or disease-severity were not imposed. The ambulatory care region within which the program operates within spans throughout the Greater Toronto Area, and expanding its reach across Ontario servicing approximately 2000 primary and secondary prevention patients over the past 4 years.

Patients began a 6-month ambulatory telemedicine-based lifestyle modification program that focuses on attaining targets for exercise, diet, cholesterol, blood pressure, diabetes, and smoking-cessation by improving health knowledge and behavior. This comprehensive lifestyle modification program guided each patient through an individualized holistic health improvement journey consisting of several integrated appointments from physicians, registered kinesiologists, and dieticians as well as comprehensive health risk assessments and risk-reports (Supplemental Appendix S1 – Programmatic Flow). The program began with the completion of a comprehensive health risk assessment to address individualized health needs and goals and finished with an exit questionnaire to determine how health behaviors and perceptions have changed over the course of the program. An integral component of the program is the vast library of over 400 educational videos and 250 podcasts organized into a set curriculum for patients and designed to improve health literacy through knowledge translation (Supplemental Appendix S2 - Top Videos and Podcasts with Engagement Metrics). Specifically with regards to cholesterol management, the program employs a cholesterol management-focused medical appointment and podcast videos to advise patients on individualized health risks and best practices to manage their cholesterol as well as to aid in patient decision-making surrounding the comprehensive management of their cholesterol.

The comprehensive lifestyle modification program operates under a continuous quality improvement learning health system (LHS) framework that allows for quality improvement and operationalizing the program in real-time. The LHS approach enhances value through optimizing impacts on patient and provider experience, population health, and health system costs.^15^ Performance benchmarks have been employed to explore feasibility and program impact on behavioral outcomes. From the program’s inception until December 15, 2020, these performance metrics were limited to self-reported exercise (weekly MET-minutes), attendance to prescheduled visits, drop-out rates, and costs to third-party payers (OHIP). In December 2020, the performance indicators expanded to include knowledge perceptions, health status, and satisfaction with care. This expansion in performance indicators coincided with the inclusion of a medication management education module on statins and antihypertensive medications.

### Patient Data

Patients referred during the study period first underwent a comprehensive health risk assessment, which included a Framingham risk score and a determination of statin eligibility based on 2021 CCS guidelines.^12^ Data obtained through the health risk assessment was supplemented through Electronic medical records to abstract additional patient data, including medication use.

Upon completion of the 6 month program, patients were administered a questionnaire at exit, which sought to explore perceptual changes in health, health knowledge, and health behaviors using a mixed open- and close-ended questionnaire.

The health risk assessment and exit questionnaires underwent in-depth content validity testing including domain determination, sampling and instrument formation.^16^ Domains of interest were identified by conducting a literature review on relevant areas such as perceptions of health status, health knowledge, health behaviors, and health-care satisfaction. Study specific questions were adapted from the Patient Global Impression of Improvement (PGI-I) index which has been previously validated as a tool to measure a patient’s perceived change in symptoms following ambulatory surgical interventions.^17-19^ Content and face validity of all questions were conducted internally by the My Heart Fitness Learning Health System clinical and research pillars. This expert panel consisted of 4 medical doctors (three cardiologists and one primary care provider), 1 nurse practitioner, 2 medical students, 2 kinesiologists, 1 dietitian, 1 pharmacist, and a community volunteer. While a content validity index score was not formally quantified, each measure was evaluated for relevance and clarity, with modifications to questionnaire items until unanimity among panelists was achieved. The questionnaires were then tested on a convenience sample of 10 patients to evaluate the feasibility of administration and the receipt of additional feedback. The instrument items were then refined and organized into a suitable format and sequence to which no further modifications were made.

This study explored perceptions among 192 consecutive patients referred between December 15, 2020 and July 2, 2021 who had completed the program and exit questionnaire. Responses were analyzed according to their statin eligibility and use as well as their self-perceived overall and mental health statuses. As a nested comparator, identical questions related to antihypertensive medication knowledge and perceptions were also administered. Open-ended questions surrounding knowledge perceptions and program feedback were also ascertained. Patient responses for program feedback were analyzed for the unprompted mention of the following key words: medication, drug, statin, prescription, cholesterol, and lipid (Supplemental Appendix S4 - Medication-Related Responses for Program Feedback).

Aggregate engagement data with program videos and podcasts was also collected through a Google Analytics platform. Number of page views, average time on page, bounce rate, and percent exit were some of the metrics collected with this platform (Supplemental Appendix S2).

### Analytic Methods

Descriptive statistics were used to analyze baseline characteristics related to the cohort on entry, and overall programmatic performance metrics throughout program. Binomial tests were used to determine whether patients’ perceptual cholesterol (and blood pressure) knowledge and medication-taking receptivity significantly changed relative to “null” as a result of the program. McNemar’s test was used to test for non-random associations between paired categorical variables within subgroup analyses (including changes in overall and mental health). A McNemar’s test was also used to determine if responses to antihypertensive medication followed the same pattern of those for statin medication (determined if there were differences on a dichotomous dependent variable between two related groups). A Pearson’s chi-squared test was applied to test variability among responses based on statin eligibility and use.

Using the thematic analysis method adapted by *Braun and Clarke* for qualitative analyses,^20^ the open-ended questionnaire responses were coded line by line. Preliminary concepts in the program feedback were identified deductively and similar concepts were grouped into themes and sub-themes. Subsequently, two other researchers conducted the same analysis and proposed themes that were discussed further to ensure that each individual coding framework captured the full range and depth of data. Any patterns and conceptual links among themes were mapped in a thematic schema. Disagreements were resolved through discussion and revisiting the data collected if needed.

Statistical significance for quantitative analyses was defined as two-tailed *P* < .05, with analyses were conducted using R^TM^ statistical software.

## Results

### Baseline Characteristics and Performance Metrics of Program Participants

**Figure 1.**
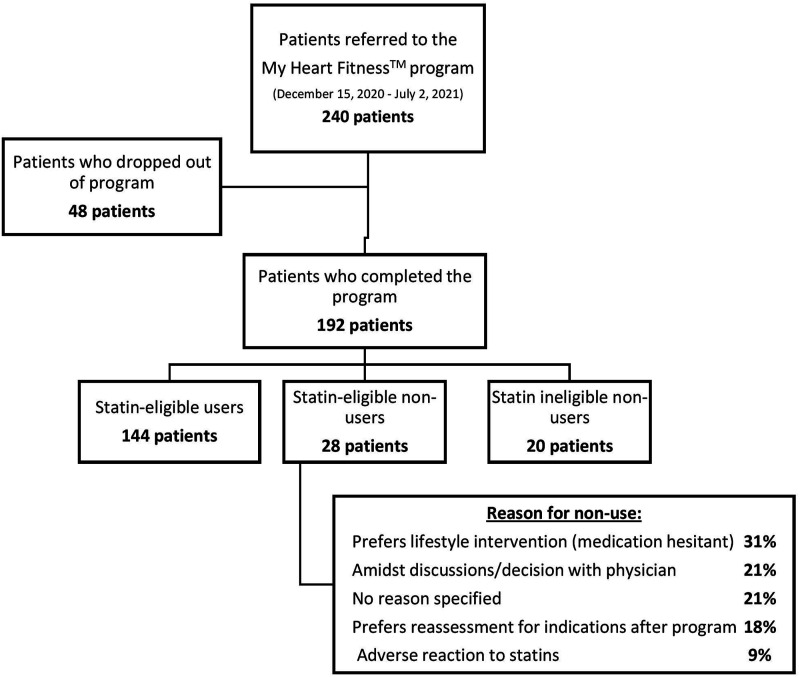
Consort diagram of My Heart Fitness patients qualifying for study and division based on their statin use and eligibility.

Between December 15, 2020 and July 2, 2021, 243 consecutive patients participated in the 6-month program, 195 of which completed the program. In total, 3 patients completed the program, but did not complete the exit questionnaire, leaving 192 patients available for analysis (79%) completed their exit questionnaires (Figure 1). Among those with completed exit interviews, the mean age was 63.68 years (SD ± 11.81); 41.15% of patients were female, 43.23% of patients had coronary artery disease, 21.35% and 53.65% of patients had diabetes or hypertension, respectively. Most participants were not meeting weight or exercise targets, 75.52% and 66.66% respectfully (Table 1). The majority of the patients were statin-eligible users (75.0%) while only a small percent were statin-eligible non-users (14.6%). Identified reasons for non-use among statin-eligible non-users included preference for lifestyle modification and medication hesitancy, ongoing discussions with patient’s physician, preference for reassessment after program and adverse reactions to statins (Figure 1).

**Table 1.** Baseline characteristics collected for 192 patients exiting the program during the study period from electronic medical records.

Variable	Sample Size (N = 192)	Mean ± SD	Frequency, %
**Demographics**			
**Age**		63.68 ± 11.81	
**Sex**			
Male	113		58.85
Female	79		41.15
**Self-reported ethnicity**			
Caucasian	43		22.40
Non-Caucasian	22		11.45
Not Reported	127		66.15
**Behavioral metrics**			
**Total MET-minutes**		704.76 ± 605.09	
≤50	37		19.27
51–400	30		15.63
401–800	61		31.77
>800	64		33.34
**Alcohol consumption**			
Within guidelines	89		46.35
Beyond guidelines	5		2.60
Does not consume	49		25.52
Not Reported	49		25.52
**Smoking status**			
Current smoker	8		4.17
Former smoker	49		25.52
No smoking history	135		70.31
**Clinical characteristics**			
**Framingham Risk score (%)**		19.0 ± 10.81	
0–0	62		32.29
10.1–20	33		17.19
>20	97		50.52
**Total cholesterol**			
1.5–3.0	20	4.11 ± 1.14	10.42
3.01–4.0	58		30.21
4.01–5.0	54		28.13
>5.01	27		14.05
Not reported	33		17.19
**LDL cholesterol**			
.5–1.50	43	2.15 ± .97	22.40
1.51–2.5	69		35.94
>2.51	49		25.52
Not reported	31		16.15
**HDL cholesterol**			
.5–1	28	1.35 ± .36	14.58
1.01–1.50	86		44.79
>1.51	47		24.48
Not reported	31		16.15
**Previous cardiac history**			
Coronary artery disease	83		43.23
**Presence of previous medical conditions**			
Diabetes	41		21.35
Pre-diabetes	22		11.46
Dyslipidemia	104		54.17
Hypertension	103		53.65
Obesity	55		28.65
**Health status**			
**Self-perceived health status**		5.38 ± 2.04	
Excellent (10)	7		3.65
Very good (7.5)	57		29.69
Good (5)	87		45.31
Fair (2.5)	38		19.79
Poor (0)	2		1.04
Not Reported	1		.52

### Change in Knowledge-Perceptions and Receptivity Towards Statin Medication

**Figure 2.**
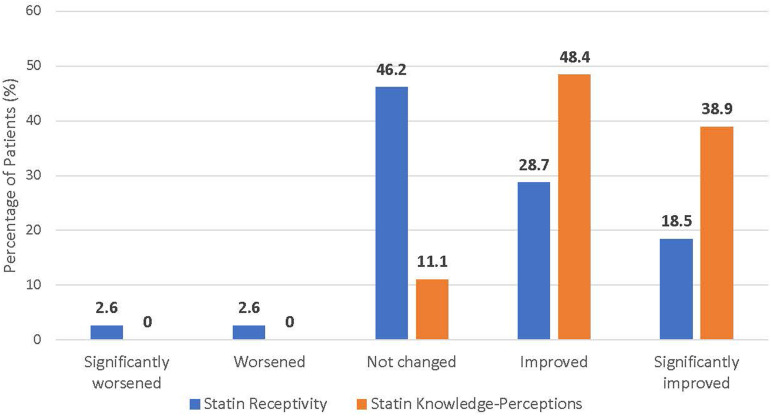
Changes in knowledge and receptivity towards statin medication was obtained for the 192 patients exiting the program through close-ended survey questions. (*P* < .0001 for statin knowledge perceptions; *P* = .61 for statin receptivity using binomial test (two-tailed)).

**Figure 3.**
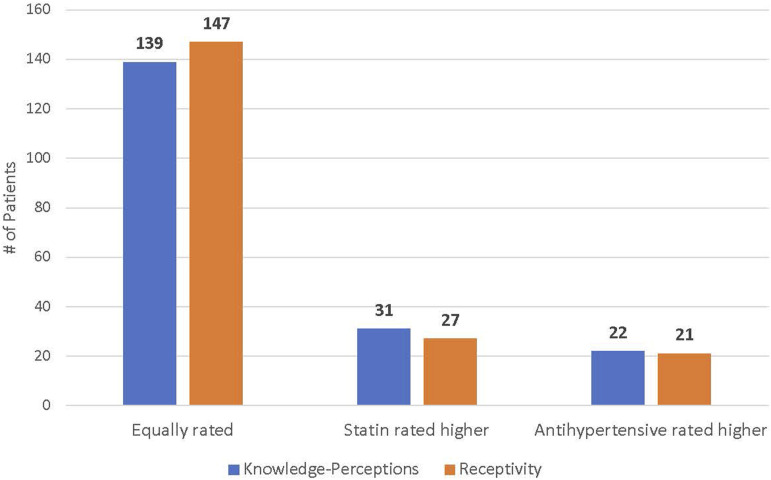
Patients’ knowledge-perceptions and receptivity towards statins and antihypertensives were relatively equal. Although more patients rated their knowledge-perceptions and receptivity towards statins higher than to antihypertensives, this difference was deemed statistically insignificant using McNemar’s test (2-tailed *P*-value for knowledge-perceptions = 1; receptivity = .28).

**Figure 4.**
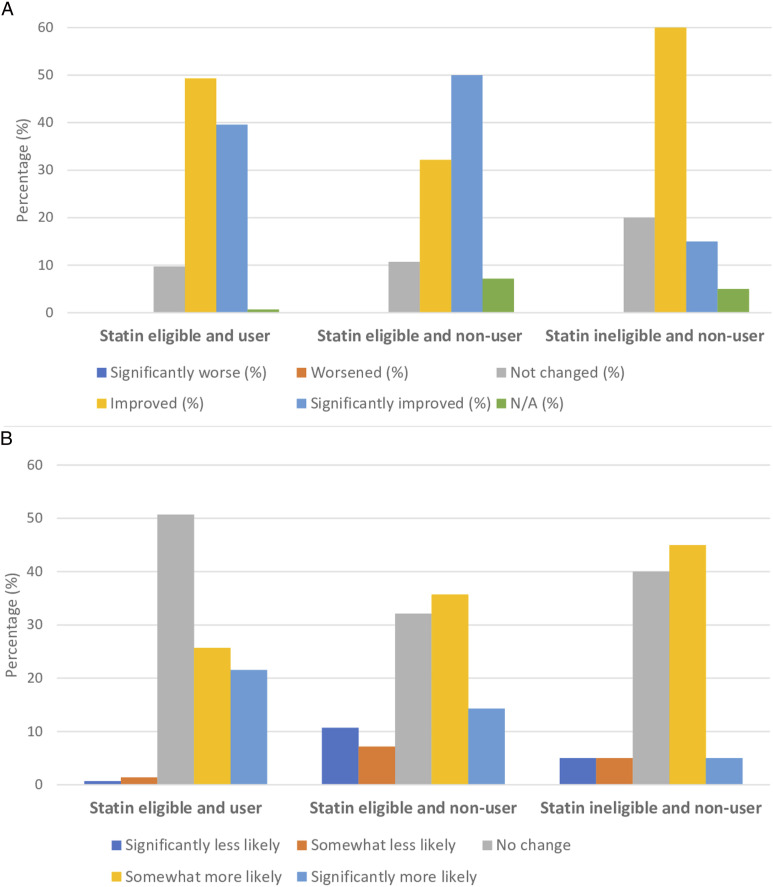
A Pearson’s chi-square test was applied to test for non-random associations between categorical variables within subgroup analyses: A. knowledge-perceptions B. receptivity. Self-perceived statin knowledge and receptivity did not vary significantly based on statin use and eligibility. For knowledge-perceptions, the chi-square statistic was 3.90 and the *P*-value was .14. For receptivity, the chi-square statistic was .11 and the *P*-value was .95.

All patients who completed the program during the study period (n = 192) were administered a mixed open- and close-ended questionnaire assessing changes in knowledge-perceptions and receptivity towards statins as well as changes in other health knowledge and behaviors. Among patients who completed the exit appointment, the response rate to the exit survey was 100%. In total, 88.4% of patients responded affirmatively to perceived improvement in cholesterol and/or statin knowledge (*P* < .0001), whereas only 48.2% of patients acknowledged that their receptivity towards taking statins increased (*P* = .61) (Figure 2). Self-perceived knowledge and receptivity towards statins did not significantly differ between those who were receiving and those who were not receiving statins, or between those patients who were or were not eligible for statins at baseline. However, self-perceived receptivity towards statins did differ significantly depending on self-perceived overall and mental health status (Table 2). Those who indicated that their self-perceived mental and overall health improved following the program were also more likely to acknowledge improvement in receptivity to statins (*P* < .0001 for overall health; *P* = .00075 for mental health). Self-perceived knowledge and receptivity towards statins also mirrored blood pressure-lowering medications, with no significant differences in responses between the two disease domains (Figure 3). Additionally, changes in knowledge-perceptions and receptivity did not significantly differ based on statin use and eligibility (Figure 4).

**Table 2.** McNemar’s Test Was Used to Test for Non-Random Associations Between Paired Categorical Variables Within Subgroup Analyses: A. Overall Health B. Mental Health. Self-Perceived Statin Receptivity Varied Significantly According to Both Self-Perceived Overall Health (*P* < .0001) and Mental Health (*P* = .00075).

	Statin receptivity worsened/no change	Statin receptivity improved	Total
**Overall health worsened/no change**	21	5	26
**Overall health improved**	79	87	166
**Total**	100	92	192
Two-tailed *P*-value = <.0001			

	Statin receptivity worsened/no change	Statin receptivity improved	Total
**Mental health worsened/no change**	47	22	69
**Mental health improved**	52	71	123
**Total**	99	93	192
Two-tailed *P*-value = .00075

### Program Outcomes and Feedback Analysis

Thematic analyses of open-ended responses identified key themes associated with the program, that may account for differences in self-perceived knowledge and receptivity towards statins. It was determined that after qualitative analyses of the first 72 patients’ open-ended responses, no new information was identified, data saturation was reached, and data collection was ceased due to the redundancy of current themes. Prevalent themes included improvements in health knowledge and awareness, promotion of positive comprehensive lifestyle behavioral modifications, and motivational and empowering support with the program’s support team (Table 3). Open-ended program feedback reaffirmed the importance of educational content in helping to shape medication management perspectives (Supplemental Appendix S4).

**Table 3.** Patient Reflections and Program Feedback Were Obtained for the 192 Patients Exiting The Program Through Open-Ended Survey Questions. The First 72 Patient Responses were Analyzed Using Thematic Analysis.

Themes	Quotations
**Improved health knowledge and awareness**	“I was able to understand the significance of exercise, diet and medications, and that helped me in following my prescriptions more diligently.” “It made me more aware about what I can do to improve my health.” “MHF is a great program that allowed me to understand my condition and how to improve my health, my life and the life of my family.”
**Positive behavioral modifications**	“It has helped me to become more disciplined and regular in exercising. I am also more conscious of my diet.” “This program helped me have healthier eating habits and I also exercised more.” “I do not like taking medication, but now I am more comfortable taking my current medication.”
**Motivational and empowering program**	“Encouraged me to exercise and eat healthy.” “It gave me more motivation.” “Thank you to My Heart Fitness for empowering me to make better decisions in living a healthier life.”
**Active communication and engagement with support team**	“I found everyone to be extremely friendly and my questions were always answered immediately or in an e-mail as soon as possible.” “It was reassuring to know that I could get answers to my questions.” “The regular check-ins answered all my questions and made me accountable to the program.”
**Comprehensive health management**	“The program taught the focus of nutrition, exercise, education and heart healthy practices.” “I was given the critical knowledge I needed to understand what I can do improve and maintain healthy habits for better heath.” “Helped me adapt a balanced diet & appropriate exercises to maintain a long-term healthy lifestyle.”
**Enhanced accountability and discipline**	“The accountability factor, which is important, is provided by regular appointments, regular exercising feedback, and adjusting and updating individual diet plans.” “It presented an accountability factor and therefore emphasized the necessary changes!” “It kept me focused. I could have otherwise given up.”
**Supportive clinic environment**	“Thanks to the entire team of MHF for doing an amazing job with dedication, diligence and above all, with CARE.” “Everyone on the team was very supportive and a pleasure to work with!” “They created and gave a caring and friendly atmosphere.” “Great group of professionals and they were my support system”

## Discussion

Our study demonstrated that the majority of patients perceived a significant improvement in their cholesterol/statin knowledge following a comprehensive lifestyle modification program. Moreover, statin receptivity rates were higher among those patients who reported improvements in their overall and mental health following completion of the program. Qualitative analyses suggest that changes in statin knowledge-perception and receptivity related to statin and antihypertensive therapy were likely attributed to successful knowledge translation efforts, comprehensiveness of care and the clinic and team environment in the program.

Our study builds on previous literature by demonstrating that a trial of therapeutic lifestyle modification may impact perceptions around knowledge and hesitancy associated with statins. Such findings reinforce the results of previous studies which have indicated that lack of proper education and failure of patients to appropriately consolidate evidence to make informed management decisions as being primary reasons for treatment gaps.^5,21^ Education through knowledge translational clinical interventions presents an opportunity to improve statin receptivity. Strategies employed in this study such as distributing educational resources and lengthening provider–patient conversations during medical appointments were shown to improve knowledge-perceptions of statins (88.4% of patients stated improvements) and could have mitigated and/or remedied statin misconceptions. This would support the 48.2% of patients who stated that their receptivity towards statins improved following the program. The findings also detailed unprompted medication-related program feedback responses which suggest that comprehensive lifestyle modification may be implicitly intertwined with medication management perspectives. Although changing receptivity towards statins among statin-eligible non-users was the main goal of this study, improving receptivity among current statin users (75% of the sample population during the study period) was also of importance, especially given evidence demonstrating that upwards of 50% of patients who are prescribed statins may discontinue therapy within one year.^22^

Questionnaire responses regarding self-perceived knowledge and receptivity towards statins remained similar irrespective of statin use or eligibility. Moreover, there were no significant self-perceived knowledge and receptivity differences between statins and antihypertensive medications, which served as an evidence-based preventative pharmacological comparator. Such results suggest that comprehensive lifestyle modification programs may have a comparatively similar influence on medication management perspectives, regardless of drug eligibility or the type of preventative pharmacotherapy examined.

Self-reported health remains a well-validated measure of health outcomes,^23^ and accordingly, serves as one of many key performance measures used to evaluate the efficacy and effectiveness of comprehensive lifestyle modification programs.^24-26^ In this study, statin receptivity was reported to be significantly higher among those individuals who also reported high overall and mental health status. Such findings are consistent with evidence demonstrating that health-seeking behaviors may be influenced by how patients perceive their own health.^6-10^ Thus, targeting ways to improve self-perceived health may be a strategy for improving health behavioral changes including changes in medication-taking behaviors.

Comprehensive health management strategies like the one administered in this study integrate several key aspects of care that can improve overall health knowledge and behaviors. Education through clinic visits and videos/podcasts delivered through a knowledge translation platform may have addressed and corrected prior misperceptions and lack of education surrounding statins and cholesterol management. Furthermore, as demonstrated through thematic analyses of open-ended responses, patients felt that the comprehensive and integrated care provided within the program, further helped them improve their knowledge and health behaviors accordingly. Such integrative, comprehensive, and holistic approaches may have facilitated a more personalized approach to knowledge translation that extended to medication management in ways that might otherwise not have been achieved with less comprehensive, individualized approaches. Moreover, available evidence suggests that patients who build trusting relationships with their providers are more likely to listen to their advice and feel confident in their management and health.^27^ As noted through thematic analyses, the supportive and motivational clinic atmosphere facilitated active communication and engagement with the multidisciplinary support team and likely built and strengthened provider-patient relationships throughout the program. Finally, patients acknowledged the importance of accountability in helping them adhere to comprehensive lifestyle management. It is possible that such accountability may have also contributed to improved perceived knowledge and/or receptivity of statin management, even among those already taking statin therapy. Our study was not designed to disentangle the relative importance of one thematic domain over others in ascribing primary reasons for perceived knowledge improvements in medication management. Rather, such findings support further research in exploring the interaction between therapeutic lifestyle management programs and medication knowledge, receptivity, and awareness.

This study may be generalizable to other multidisciplinary therapeutic lifestyle and preventative programs that incorporate patient education and exercise counseling. For example, as with MHF, cardiac rehabilitation programs also offer educational counseling, goal setting, motivational interviewing, and exercise prescriptions in accordance with ACSM guidelines. That being said, differences between MHF and other preventative programs, such as routine integration of telemedicine physician visits, may have resulted in a more integrated clinical care experience with MHF as compared with other preventative programs devoid of routine physician visits. Future research will be needed to better evaluate comparative effectiveness between MHF and other preventative care programs including cardiac rehabilitation.

As a means for continuous quality improvement of the program, the comprehensive lifestyle modification program operates under a learning health system framework where each learning cycle consists of implementing new interventions, monitoring outcomes, and informing program operations. Following the implementation of medication management education efforts this cycle, several improvements in programming have been identified and mainly focus on adopting a more comprehensive approach to managing patient opinions and decisions surrounding medication by acting as a decision aid. Decision aids integrate patient values into options, benefits, risks, and uncertainties associated with different therapies to help guide patient decisions.^28^ Adding a decision aid to current educational interventions in the program may allow for improved knowledge, decisional conflict, and medication management for those hesitant to take statins.^29^ Other modifications to programmatic implementation being explored as a result of our study include the incorporation of the Modified Morisky Scale (MMS) to ascertain medication adherence as well as medication management tracking and prescription refills.^30^ In short, in response to this study, the MHF LHS pillar stakeholders felt that knowledge and education alone may not be sufficient to change hesitancy around medication management. Ultimately, if patients still refuse statins, decision aids might better direct patients to alternative recommendations. Other counseling approaches such as the transtheoretical model of behavior change and motivational interviewing might then be implemented to try and improve receptivity and health behaviors.^31,32^

Several limitations exist in the study. First, this study was underpowered and lacked a control group which makes it difficult to draw meaningful conclusions from the findings and make relevant subgroup comparisons. The potential for sample bias is also a limitation as patients referred to the program were mainly at-risk primary and secondary prevention patients and were likely looking to improve health knowledge, behaviors, and perceptions. Baseline health literacy levels, which may impact the generalizability of the study, were not formally examined. Response bias also could have been present as only one questionnaire was administered at the end of the program. Future research will include pre- and post-program questionnaires to mitigate this effect and will consider the potential benefits of evaluation throughout as well. Lastly, the exit questionnaire evaluated self-reported knowledge-perceptions rather than actual knowledge which could be corrected by implementing knowledge-testing tools into the program. Statin knowledge-perceptions and receptivity could have also been impacted by the nocebo effect which has been previously observed in studies involving statin users.^33^ As the study was conducted during the COVID-19 global pandemic, this could have had an effect on participation and drop-out rates. The extent to which the pandemic may have impacted the results of this study is unclear. However, based on our data, engagement metrics such as attendance to prescheduled telemedicine visits did not significantly vary as compared to pre-pandemic periods. Regardless of these limitations, the study was designed to explore feasibility and generate hypotheses. Further research confirming the generalizability of our findings and examining the efficacy of the program on mitigating statin treatment gaps through pragmatic clinical trials are required.

In conclusion, our study demonstrates that patients’ perceived knowledge and receptivity towards statins may improve following their participation in a comprehensive therapeutic lifestyle program. These results underscore the need for future research, to not only evaluate the impact of theraeputic lifestyle modification programs on statin uptake, compliance and outcomes, but also to explore more integrative comprehensive approaches to medication management decision-making when patients hesitancies towards evidence-based preventative pharmacotherapies exist.

## Supplemental Material

Supplemental material - Evaluating Statin Knowledge-Perceptions and Receptivity Following a Comprehensive Lifestyle Modification Program Supplemental material for Evaluating Statin Knowledge-Perceptions and Receptivity Following a Comprehensive Lifestyle Modification Program by Alaina Pupulin, Jillian Ball, Ravi Bajaj, David A. Alter in American Journal of Lifestyle Medicine
