# Supplementary material for: Evaluating Statin Knowledge-Perceptions and Receptivity Following a Comprehensive Lifestyle Modification Program
Source: Am J Lifestyle Med. 2023 Mar 16;20(3):405–17. doi: 10.1177/15598276231163129 (PMC12864696; doi:10.1177/15598276231163129)
Supplement: Supplemental material - Evaluating Statin Knowledge-Perceptions and Receptivity Following a Comprehensive Lifestyle Modification Program [file sj-pdf-1-ajl-10.1177_15598276231163129.pdf]

Supplemental Appendix S1 – Programmatic Flow

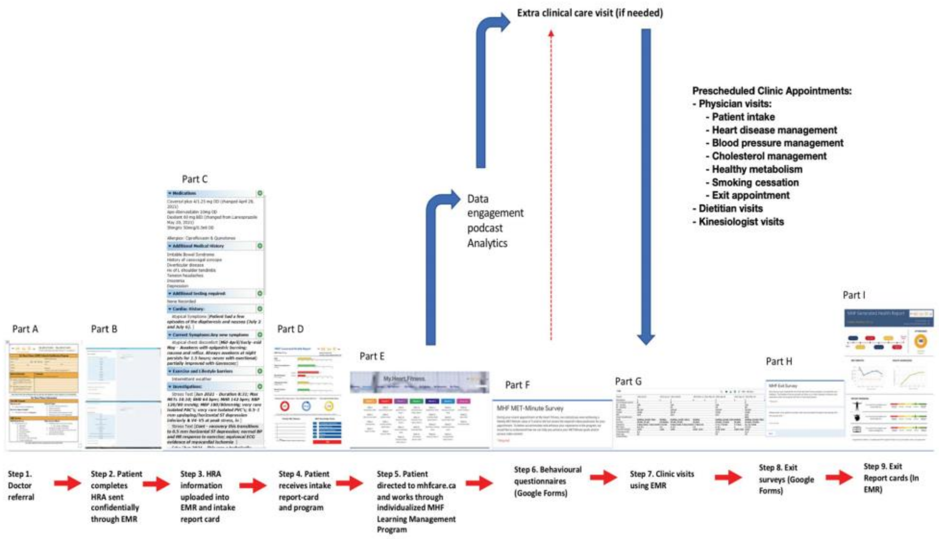

Step 1: All patients who participate in MHF must require a referral by either their primary care or specialty care provider. Eligible patients are any adult (>18) who is felt to be in need of a preventative lifestyle program because they are deemed at risk for

cardiovascular disease (one or more vascular risk-factors) or already have established vascular disease (cardiovascular, peripheral vascular, or cerebrovascular disease). Given the broad applicability of prevention and educational counseling for lifestyle modification, we have not imposed exclusion criteria, but rather have only restricted access to patients ages 18 years or older. Our rationale for age restriction is due to the fact that our care-providers manage adult populations only. See Part A for referral form.

Step 2: All patients are then directed by MHF to complete a Health Risk Assessment (HRA). The HRA includes ethno demographic, clinical, and behavioural factors that help determine the type of educational content, the frequency of clinical visits, and the duration of follow-up. The HRA is sent directly from the patient's Electronic Medical Record (EMR). See Part B for health risk assessment.

Step 3: Once patients complete their HRA, the HRA responses are inputted into EMR by the clinical support/administrative team. See Part C (screenshot of EMR 'test' patient).

Step 4: Patient's intake (first clinical appointment) includes a health risk report card, which is generated from the information provided from the EMR. The report card is given to the copy and to the referring provider. See Part D (Health intake report card).

Step 5: Patient is directed to educational content (podcasts/video) by the health care team. Patient's access the educational content through a password-protected knowledge platform web-portal. Data stored on web-portal is PHIPA compliant. Within the platform web-portal, patients access provider-directed educational content. The specific educational content reflects a patient's risk and behavioural profile, his/her exercise and dietary educational needs, and self-management skill requirements. See Part E (screenshot of web-portal).
MHF tracks engagement with said educational content through google analytics. Data analytics are according to study ID (file number) and not by personal identifiers. The data analytics are used in part to generate additional provider clinical visits. For example, patients who do not complete review of prescribed educational content may result in one of the following clinical responses: (1) An automated reminder message; (2) A telephone call from our support/administrative team; (3) A clinic visit. The clinical response is also dependent on the intermediary behavioural engagement surveys below (step 6).

Step 6: Patient completes intermediary behavioural engagement surveys, exercise (MET-MINUTE) surveys, anthropometric data. This data is uploaded into google charts. No personal identifiers exist within any of the survey data collected. The data is stored

according to the patient's file ID number. Intermediary behavioural engagement survey responses in addition to the Google data analytics noted above determine the clinical responses (automated reminder; telephone calls; clinic visit) – See Part F for behavioural engagement, MET-Minute/Anthropometric surveys.

Step 7: Support/administrative team abstract behavioural metrics from the intermediary behavioural engagement surveys and enter such data into the EMR charts for on-going clinical follow-up. See Part G (EMR screenshot of hypothetical patient's data). Throughout the course of the 6-month program, patients will undergo several clinic visits by physicians, kinesiologists and dietitians. Each month, patients have dedicated physician visits where the following areas are discussed and targeted: heart disease, blood pressure management, cholesterol management, metabolism, and smoking cessation. Patients' health and performance indicators are recollected at each of these visits to track program progress and patients are prescribed tasks for their next visits. All patient questions, comments and concerns are answered at these visits by health providers.

Step 8: Patient completes exit survey questionnaire at 6 months following program entry (Part H). Data elements are used to generate exit clinical report cards for patients and referring providers (Part I).

Data Sources

1. Learning Management System (LMS) - All patient questionnaire data required for patient care, including the health risk assessment, intermediary (mid-point) and exit questionnaires, health knowledge quizzes, self-management goal-setting and monitoring tasks are housed within the MHF LMS. Access into the MHF LMS requires a username and password. All data stored within the LMS is encrypted.

2. Electronic Medical Records (EMR) - Given that the LMS is not an Ontario Ministry approved EMR, physicians still document all clinical interactions through an EMR. Similarly, lab work, diagnostic testing, medication prescriptions, physician reporting, and billing are conducted through EMR. ACCURO EMR (™) is used as the EMR for the purposes of patient-provider MHF telemedicine clinic, ACCURO EMR on a secured server following all requirements set out by PHIPA (Personal Health Information Protection Act) [2]. While there are redundancies in clinical data between the LMS and EMR (such as patient anthropometric measurements and recorded vitals (including heart rate, blood pressure, and weight), health risk assessment forms, and weekly MET-MINUTE data, the information is often abstracted from one source (the LMS) into the other (EMR) by our support team when prepping for individual

patient clinics. The EMR allows us to evaluate clinical information within the context of an individual clinical interaction, whereas the LMS does not contain data related to the clinical encounter itself. Accordingly, the EMR provides such information as pre-scheduled visit attendance, self-reported MET-MINUTES per week, and other anthropometric values blood pressures, weight, height, BMI, and waist circumference at each patient encounter. Our rationale for distinguishing the EMR and LMS is that other health systems may wish to similarly utilize their own EMR system when integrating the LMS into their health-care system. While the EMR requires data obtained through the LMS to facilitate a clinical interaction, the two sources will be kept distinct for the foreseeable future. As a result, data related to the clinical interaction itself will not be housed within the LMS.

3. Web Engagement Analytics - All information related to our video and podcast content is available in Google Analytics (which is essentially part of the website). Aggregate data regarding average video watch time, most watched video, least watched video, device used to watch video, and most active times of watching are all metrics extracted and used to better inform our content quality over time. The same metrics apply for our podcasts. All content metrics are used for QI purposes at MHF within the LMS.

Supplemental Appendix S2 - Top Videos and Podcasts with Engagement Metrics							
Number	Page	Pageviews	Unique Pageviews	Avg. Time on Page	Entrances	Bounce Rate	% Exit
1	/videos-md-series/	526	346	0:05:38	261	60.31%	52.09%
2	/videos-introduction/	242	174	0:04:54	124	57.38%	49.59%
3	/videos-resistance-training/	239	154	0:03:05	60	62.71%	41.84%
4	/videos-kinesiology/	176	138	0:07:42	82	66.67%	53.41%
5	/podcast/d116-metabolism/	161	137	0:07:40	116	79.31%	72.05%
6	/videos-registered-dietitian/	152	116	0:05:06	46	47.83%	47.37%
7	/podcast/c003-what-hypertension-feels-like/	118	100	0:05:01	54	50.00%	51.69%
8	/podcast/c009-statins/	95	82	0:10:25	49	56.25%	48.42%
9	/videos-balance/	89	50	0:02:17	21	61.90%	41.57%
10	/podcast/k012-importance-of-resistance-training/	83	73	0:05:52	33	60.61%	59.04%
11	/podcast/k025-exercise-vs-physical-activity-part-2/	68	53	0:06:43	24	37.50%	35.29%
12	/stretching-videos/	67	57	0:03:01	26	71.43%	61.19%
13	/aerobic-cardio-videos/	61	43	0:01:43	14	64.29%	31.15%
14	/podcast/d077-statins-and-grapefruit/	51	45	0:08:29	36	47.22%	49.02%
15	/podcast/k003-physical-activity-vs-exercise/	44	39	0:08:56	26	53.85%	47.73%
16	/podcast/k005-moderate-exercise/	42	36	0:09:06	24	66.67%	66.67%
17	/podcast/d018-dietitians-vs-nutritionist/	41	35	0:05:57	26	46.15%	36.59%
18	/podcast/d115-dietary-cholesterol-and-impacts-on-blood-cholesterol/	41	35	0:06:42	10	60.00%	36.59%
19	/podcast/p004-sleep-remedies/	39	33	0:06:39	25	64.00%	66.67%
20	/videos-podcast-pacing/	32	21	0:03:29	5	80.00%	21.88%
21	/podcast/k019-tips-on-exercising-safety/	30	27	0:05:18	20	70.00%	60.00%
22	/podcast/d078-food-and-sleep/	26	26	0:10:39	14	57.14%	73.08%
23	/podcast/d045-measuring-success-outside-of-the-scale/	22	9	0:02:34	2	0.00%	9.09%
24	/podcast/d114-dash-diet/	19	17	0:06:32	8	87.50%	73.68%
25	/podcast/d026-nutrition-smart-goals/	15	12	0:03:27	3	33.33%	26.67%
26	/podcast/d012-positive-framing-for-dieting/	11	10	0:03:57	0	0.00%	18.18%
27	/podcast/d016-eating-together/	11	4	0:00:57	1	0.00%	9.09%
28	/podcast/d024-eggs-and-cholesterol/	11	10	0:07:04	1	100.00%	54.55%
29	/podcast/c008-exercise-safety-and-arrhythmias/	10	9	0:07:14	2	50.00%	30.00%
30	/podcast/k017-what-is-podcast-pacing/	8	8	0:17:04	4	100.00%	62.50%
31	/podcast/k020-tracking-progress-importance-of-tracking-exercise/	8	6	0:07:57	1	0.00%	12.50%
32	/podcast/p016-natural-health-products/	8	7	0:06:01	5	20.00%	37.50%
33	/scheduler-wrap-up-month/	8	5	0:09:15	0	0.00%	12.50%
34	/podcast/d005-olive-oil/	7	2	0:01:09	0	0.00%	28.57%
35	/podcast/d040-soluble-fibre/	7	7	0:03:55	4	66.67%	57.14%
36	/podcast/k029-aerobic-exercise-for-the-heart/	7	6	0:02:00	0	0.00%	14.29%
37	/podcast/n001-sleep/	7	6	0:03:43	3	66.67%	42.86%
38	/podcast/p002-sleep-interventions/	7	6	0:06:15	5	25.00%	14.29%
39	/podcast/k026-exercise-is-medicine/	6	4	0:03:39	0	0.00%	16.67%

40	/podcast/c004-ways-to-reduce-blood-pressure-without-medications/	5	5	0:10:48	1	0.00%	0.00%
41	/podcast/d047-keto-diet/	5	4	0:00:48	0	0.00%	40.00%
42	/podcast/d061-dietitian-discovery-clinic-visit/	5	5	0:02:36	0	0.00%	20.00%
43	/podcast/d084-best-weight-principle-and-healthy-eating-lifestyle/	5	5	0:05:43	0	0.00%	40.00%
44	/podcast/k006-exercising-in-the-heat/	5	5	0:03:21	0	0.00%	40.00%
45	/podcast/d015-food-diaries-self-tracking/	4	3	0:02:27	0	0.00%	0.00%
46	/podcast/d037-nutrition-food-recording-apps/	4	4	0:03:41	0	0.00%	25.00%
47	/podcast/d100-sodium/	4	4	0:07:43	0	0.00%	25.00%
48	/podcast/d105-butter-vs-margarine/	4	3	0:04:54	0	0.00%	50.00%
49	/podcast/k009-met-minutes/	4	4	0:03:41	1	0.00%	25.00%

**DATE**	MARCH 01 2021 - DECEMBER 15 2021
**DATA SOURCE**	GOOGLE ANALYTICS - AUDIENCE - SITE CONTENT - ALL PAGES
**DATA OVERVIEW**	This data is a list of the most common pages* by most page views. We are looking at data to see which videos and podcasts are most popular, i.e. have the most page views**.
**NOTES**	*The pages visited, listed by URI. The URI is the portion of a page's URL following the domain name; for example, the URI portion of www.example.com/contact.html is /contact.html.
	**Pageviews is the total number of pages viewed. Repeated views of a single page are counted.
**Definitions**	**Entrances**: Entrances is the number of times visitors entered your site through a specified page or set of pages.
	**Bounce rate**: The percentage of single-page sessions in which there was no interaction with the page. A bounced session has a duration of 0 seconds.
	**% Exit**: %Exit is (number of exits) / (number of pageviews) for the page or set of pages. It indicates how often users exit from that page or set of pages when they view the page(s).
	**Unique page views**: Unique Pageviews is the number of sessions during which the specified page was viewed at least once. A unique pageview is counted for each page URL + page Title combination.
**Limitations**	While podcasts have their own unique page url, unfortunately, videos do not because they open as a youtube pop-up on the website. This means that we can track the category of videos that have the highest page views, but we cannot see which videos are most popular.

Supplemental Appendix S3 - Exit Questionnaire

MHF Exit Survey

Now that you have reached the end of the My Heart Fitness program, we would like your feedback. The feedback that you provide will allow us to make changes to enhance your experience within the program and that of future participants.

* Required

1. Please enter your patient number that was provided through email along with this survey link. *

____

Skip to question 2

Health

2. How would you rate your overall health? *

Mark only one oval.

- ◯ Poor
- ◯ Fair
- ◯ Average
- ◯ Very good
- ◯ Excellent

3. How would you rate your mental health? *

Mark only one oval.

- ◯ Poor
- ◯ Fair
- ◯ Average
- ◯ Very good
- ◯ Excellent

4. As a result of our program, my health knowledge has... *

Mark only one oval.

- Significantly worsened
- Worsened
- Not changed
- Improved
- Significantly improved

5. As a result of our program, my health has... *

Mark only one oval.

- Significantly worsened
- Worsened
- Not changed
- Improved
- Significantly improved

6. As a result of our program, my mental health has... *

Mark only one oval.

- Significantly worsened
- Worsened
- Not changed
- Improved
- Significantly improved

Skip to question 7

Exercise

7. How do you feel our program has influenced your exercise habits? Since starting the program, I am exercising… *

Mark only one oval.

- Significantly less *Skip to question 8*
- Somewhat less *Skip to question 8*
- No change *Skip to question 8*
- Somewhat more *Skip to question 9*
- Significantly more *Skip to question 9*

Exercise Explanation

8. If your exercise frequency has not changed or decreased, please explain why. *

Diet

9. Since starting the program, I am eating vegetables and fruit (fresh, frozen, canned)… *

Mark only one oval.

- Significantly less
- Somewhat less
- No change
- Somewhat more
- Significantly more

10. Since starting the program, I am eating whole grains (whole grain bread/pasta, quinoa, barley, brown rice, oatmeal, popcorn)... *

Mark only one oval.

- Significantly less
- Somewhat less
- No change
- Somewhat more
- Significantly more

11. Since starting the program, I am eating plant-based proteins/healthy fats (beans, lentils, tofu/soy, nuts, seeds, fish, olive oil)... *

Mark only one oval.

- Significantly less *Skip to question 12*
- Somewhat less *Skip to question 12*
- No change *Skip to question 12*
- Somewhat more *Skip to question 13*
- Significantly more *Skip to question 13*

Diet Explanation

12. If your frequency of making healthy food choices has not changed or decreased, please explain why. *

Skip to question 13

Medications

13. To what extent do you feel the program has influenced your level of knowledge about cholesterol-lowering medications? My level of knowledge has... *

Mark only one oval.

- Significantly worsened
- Worsened
- Not changed
- Improved
- Significantly improved
- Not applicable

14. To what extent do you feel the program has influenced you level of knowledge about blood pressure-lowering medications? My level of knowledge has... *

Mark only one oval.

- Significantly worsened
- Worsened
- Not changed
- Improved
- Significantly improved
- Not applicable

15. Based on what you've learned from the program, which of the following statements regarding receptivity towards cholesterol-lowering medication best applies to you? *

Mark only one oval.

- I am significantly less likely to take cholesterol-lowering medication following the program
- I am somewhat less likely to take cholesterol-lowering medication following the program
- There is no change in my likelihood of taking cholesterol-lowering medication
- I am somewhat more likely to take cholesterol-lowering medication following the program
- I am significantly more likely to take cholesterol-lowering medication following the program

16. Based on what you've learned from the program, which of the following statements regarding receptivity towards blood pressure lowering medication best applies to you? *

Mark only one oval.

- I am significantly less likely to take blood pressure lowering medication following the program
- I am somewhat less likely to take blood pressure lowering medication following the program
- There is no change in my likelihood of taking blood pressure lowering medication
- I am somewhat more likely to take blood pressure lowering medication following the program
- I am significantly more likely to take blood pressure lowering medication following the program

Skip to question 17

Program Satisfaction

17. How would you rate your satisfaction with the program? *

Mark only one oval.

- Very dissatified
- Dissatisfied
- Neutral
- Satisified
- Very satisfied

18. How likely would you be to recommend this program to your family or friends? *

Mark only one oval.

- Very unlikely
- Unlikely
- Neutral
- Likely
- Very likely

19. How did your understanding about the importance of physical activity and exercise change as a result of this program? *

20. How did our program help you improve your health? *

21. What did you like least about the program, and what suggestion would you make to help improve the program? *

22. What could we have done for you to be more invested in the program (i.e showing up to appointments, regularly exercising, adjusting your diet)? *

23. Much of the costs of the program were not eligible for reimbursement from OHIP. Such costs were paid for on your behalf by My Heart Fitness in order to serve and improve the health of the community. Would you have been willing to pay out of pocket for this program? If so, how much? Would you be willing to provide a financial contribution (of any amount of your choosing) to help sustain the program?

24. Would you provide us with a testimonial to post on the website and marketing materials? If so, please provide your testimonial below and indicate if you would like your testimonial to be anonymous.

25. Please provide any additional comments.

This content is neither created nor endorsed by Google.

Supplemental Appendix S4 – Medication-Related Responses for Program Feedback

"I was able to understand the significance of exercise, diet and medications, and that helped me in following my prescriptions more diligently."
"Easy to understand educational videos about heart health, cholesterol and hypertension."
"Explaining cholesterol and how to maintain good cholesterol."
"I do not like taking medication but now I am more comfortable taking my current medication."
"Kept me motivated and increased knowledge level regarding cardiac-related diseases."
"The occasional videos explaining cholesterol and plague accumulation were very informative and quite well done. They made it easier to understand the message being conveyed."
"My health, especially with regard to cholesterol and hypertension, improved dramatically after joining MHF."
"The most important factor, in my opinion, is the regularly scheduled appointments with the various cardiologists to answer questions, update research information on drugs, discuss various alternatives and reduce the fear of the unknown which causes such enormous stress!"
"In every way possible. Pushing my knowledge boundaries in terms of exercise, diet, medications, and cholesterol. It enhanced and motivated what I already knew."
"Periodic interactions [with providers] helped me educate on compliance to the regular exercise, diet & medications in improving heart health."
"[Improved] knowledge of medications."
"[The program helped me] keep up with my medications and exercise."
"It gave me a clearly understanding about my heart, diet, exercise and medication."
"It just wasn't about exercise, it's about diet, it's about understanding how your medication works for you, how to minimize the risks in your life and maximize the benefits.<br>Those questions were answered fast because it was so easy to ask questions at any time.<br>I don't feel this program should be an option. It should be mandatory. The knowledge I received in this program helps me live a more active and healthier lifestyle."
"The MHF program has provided me with in depth knowledge about heart diseases, cholesterol, blood pressure and how diet and exercise can have a dramatic positive impact on a person's health."
"More aware of how food and medications can affect my health (both positively and negatively)."
"You did everything, and I followed the prescriptions. [I] got my blood pressure in very good control."
'Being made aware of how serious high cholesterol has made me take measures to do my part to get that under control."

Appendix I: Patient responses for program feedback were analyzed for the unprompted mention of particular key words: medication, drug, statin, prescription, cholesterol and lipid.
